# ^23^Na Magnetic Resonance Imaging of the Lower Leg of Acute Heart Failure Patients during Diuretic Treatment

**DOI:** 10.1371/journal.pone.0141336

**Published:** 2015-10-26

**Authors:** Matthias Hammon, Susan Grossmann, Peter Linz, Christoph Kopp, Anke Dahlmann, Christoph Garlichs, Rolf Janka, Alexander Cavallaro, Friedrich C. Luft, Michael Uder, Jens Titze

**Affiliations:** 1 Department of Radiology, University Hospital Erlangen, Erlangen, Germany; 2 Department of Nephrology and Hypertension, University Hospital Erlangen, Erlangen, Germany; 3 Department of Cardiology, University Hospital Erlangen, Erlangen, Germany; 4 Experimental and Clinical Research Center, Charité Medical Faculty and the Max Delbrück Center for Molecular Medicine, Berlin, Germany; 5 Department of Medicine, Vanderbilt University, Nashville, Tennessee, United States of America; Northwestern University Feinberg School of Medicine, UNITED STATES

## Abstract

**Objective:**

Na^+^ can be stored in muscle and skin without commensurate water accumulation. The aim of this study was to assess Na^+^ and H_2_O in muscle and skin with MRI in acute heart failure patients before and after diuretic treatment and in a healthy cohort.

**Methods:**

Nine patients (mean age 78 years; range 58–87) and nine age and gender-matched controls were studied. They underwent ^23^Na/^1^H-MRI at the calf with a custom-made knee coil. Patients were studied before and after diuretic therapy. ^23^Na-MRI gray-scale measurements of Na^+^-phantoms served to quantify Na^+^-concentrations. A fat-suppressed inversion recovery sequence was used to quantify H_2_O content.

**Results:**

Plasma Na^+^-levels did not change during therapy. Mean Na^+^-concentrations in muscle and skin decreased after furosemide therapy (before therapy: 30.7±6.4 and 43.5±14.5 mmol/L; after therapy: 24.2±6.1 and 32.2±12.0 mmol/L; *p*˂0.05 and p˂0.01). Water content measurements did not differ significantly before and after furosemide therapy in muscle (*p* = 0.17) and only tended to be reduced in skin (*p* = 0.06). Na^+^-concentrations in calf muscle and skin of patients before and after diuretic therapy were significantly higher than in healthy subjects (18.3±2.5 and 21.1±2.3 mmol/L).

**Conclusions:**

^23^Na-MRI shows accumulation of Na^+^ in muscle and skin in patients with acute heart failure. Diuretic treatment can mobilize this Na^+^-deposition; however, contrary to expectations, water and Na^+^-mobilization are poorly correlated.

## Introduction

Acute heart failure involves Na^+^ accumulation (as Cl^-^) putatively in the extracellular space [1]. We observed that Na^+^ is deposited in skin and muscle physiologically and more so in hypertension [2–4]. The Na^+^ is bound to negatively charged glycosaminoglycans (GAG). A very local hypertonic (to plasma) increase in Na^+^ concentrations leads to stimulation of tonicity element binding protein (TonEBP) transcription factor in monocyte phagocytic system (MPS) cells [5–7]. TonEBP stimulates vascular endothelial growth factor-C (VEGF-C) production that stimulates lymphatics. An increase in lymph-capillary density regulates this site of Na^+^ storage. The relevance of this regulatory system to salt-sensitive hypertension was demonstrated previously [5–7]. Furthermore, long-term balance studies documented infradian rhythms in Na^+^ balance and excretion that are highly consistent with an additional Na^+^ storage compartment [8]. Carcass ashing and atomic absorption spectrometry were used, clearly methods that have no human application [2–7]. Using sodium (^23^Na) as a target for molecular (atomic) imaging is not new [9]. The heart has recently been suggested as a target [10]. Cardiac and other applications for Na^+^ magnetic resonance imaging (^23^Na-MRI) have been reviewed [11–13]. Zaaraoui et al. showed that the distribution of brain sodium accumulation correlates with disability in multiple sclerosis [14]. Ouwerkerk et al. found an increased Na^+^ concentration in malignant breast tissue [15]. Other studies concerned osteoarthritis [16] and indicated that ^23^Na-MRI has the potential to provide insight into muscle physiology [17–23]. Our aim was to determine whether the Na^+^ storage depot we described could be visibly reduced after therapy for acute heart failure. Patients with acute heart failure undergo rapid volume shifts during therapy. We selected these patients for further study of ^23^Na-MRI. We hypothesize that the behavior of the Na^+^ storage depot could have a long-term influence on outcomes for patients with cardiovascular disease [24].

To test our hypothesis regarding the mobility of Na^+^ stores, we recruited acute heart failure patients and normal subjects after we had developed ^23^Na-MRI for our clinical purpose [25, 26]. We developed a coil for ^23^Na-MRI utilizing the upper calf as a target that allows skin, skeletal muscle, and bone to be investigated. In earlier studies, we validated the utility of this method in experimental animals and human subjects, including hemodialysis patients [27].

## Materials and Methods

### Patient recruitment

The internal review board of the University Hospital Erlangen, Germany, approved the study (Re.-No. 3948). All participants gave their written informed consent. The study was conducted according to the principles of the Declaration of Helsinki.

Patients with acute heart failure were prospectively recruited from the cardiology service and the emergency department. We selected patients who were able to give informed consent, were able to clinically tolerate the procedure, including being supine for 30 min, and whose physicians cleared them for the study. We were able to match the patients with normal controls in terms of age and sex. Also obviously excluded were persons with metal implants or claustrophobia. From January to September 2012, we included patients who were scheduled to receive intravenous furosemide, based on their physician’s clinical judgment, and who gave their written informed consent. Patients underwent ^23^Na-MRI immediately before and after the guidelines-oriented intravenous furosemide therapy. Normal volunteers were recruited by advertisement and underwent ^23^Na-MRI once. All subjects underwent medical examinations and ^23^Na-MRI in our clinical research center.

### Experimental setup and imaging technique

^23^Na-MRI for quantitative analysis in man was implemented and the methods were validated and recently published [25–27]. Na^+^ content was measured in lower leg muscle and skin (at the level of the largest circumference) with a custom-made ^23^Na knee-coil (Stark-Contrast, Erlangen, Germany) at 3.0 T with a magnetic resonance imaging scanner (Magnetom Verio, Siemens Healthcare, Erlangen, Germany) before and after diuretic therapy using a gradient echo ^23^Na sequence (total acquisition time TA: 3.25 minutes, echo time TE: 2.07 ms, repetition time TR: 100 ms, flip angle FA: 90°, 32 averages, resolution: 3 x 3 x 30 mm^3^). The gradient echo ^23^Na sequence was acquired four times successively.

In parallel, water content was quantified in tissue by ^1^H-MRI, using a fat-suppressed inversion recovery sequence with spin density contrast (total acquisition time: 6.22 minutes, inversion time TI: 210 ms, echo time TE: 12 ms, repetition time TR: 3000 ms, flip angle FA1/2: 90°/180°, resolution: 1.5 x 1.5 x 5 mm^3^), as described by other investigators [8]. A T1-weighted fast-low-angle-shot (FLASH)-sequence was acquired to depict the anatomy and morphology of the lower leg. The scanning protocol is shown in Table 1.

**Table 1:** Scanning protocol.

	Localizer	T1-weighted fast-low-angle-shot (FLASH)-sequence	Fat-suppressed inversion recovery sequence	Gradient echo ^23^Na sequence (acquired 4 times successively)
***Total acquisition time (TA; min)***	0.15	2.08	6.22	3.25
***Echo time (TE; ms)***	4	2.46	12	2.07
***Repetition time (TR; ms)***	8.6	250	3000	100
***Inversion time (TI; ms)***	-	-	210	-
***Flip angle (FA; °)***	20	60	90/180	90
***Averages***	2	2	1	32
***Bandwidth (Hz/pixel)***	320	310	130	430
***Field of view (FoV; mm)***	192	192	192	192
***Matrix (pixel)***	256	256	128	64
***Resolution (mm)***	0.75 x 0.75 x 10	0.75 x 0.75 x 5	1.5 x 1.5 x 5	3 x 3 x 30

### Image analysis

**Fig 1:**
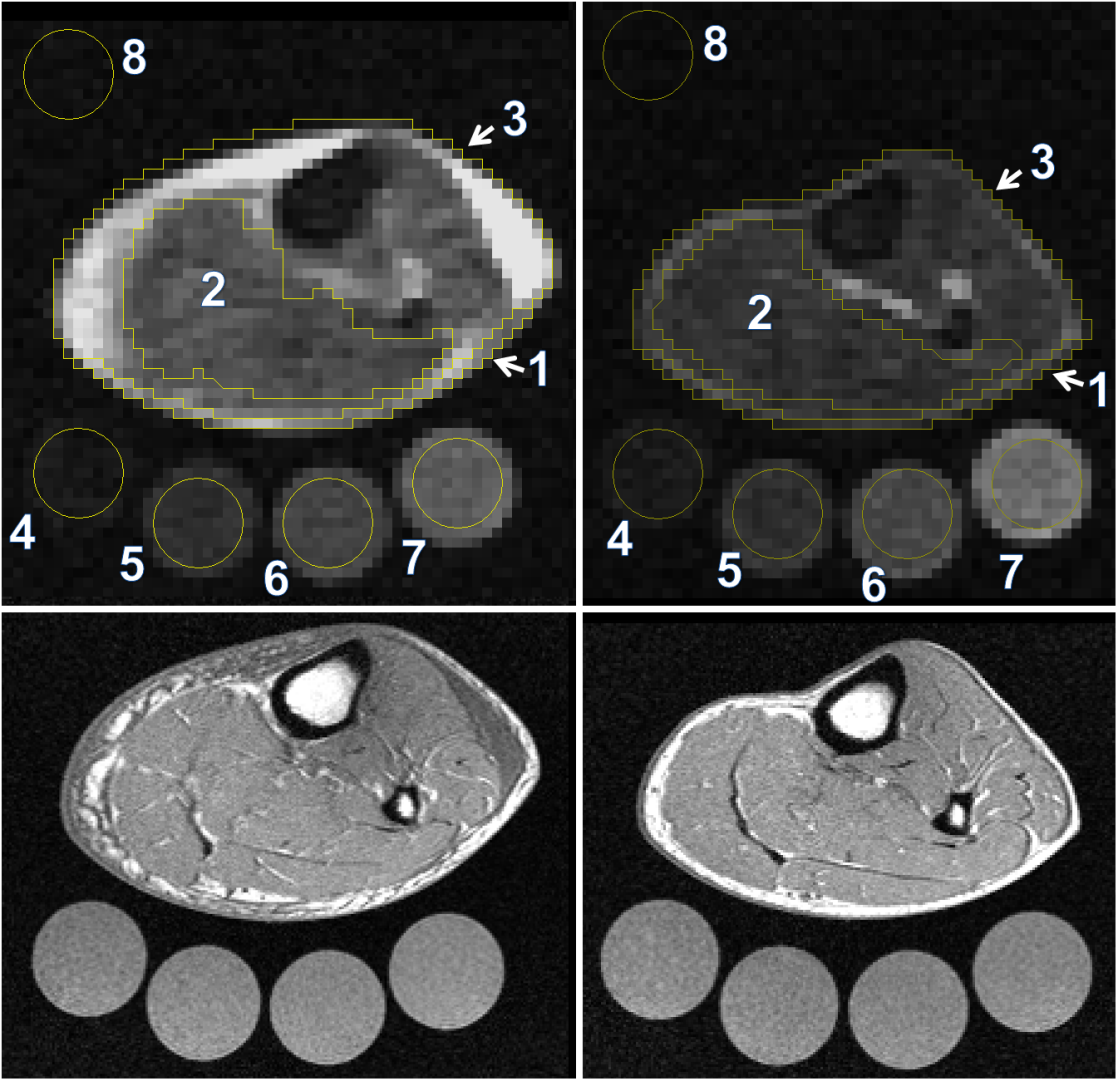
Evaluation of the compartments of the lower leg. The compartments of the lower leg were determined before (left) and after (right) diuretic therapy by an experienced reader. The different anatomical regions of interest (ROI) were drawn guided by the anatomical image (lower images, T1-weighted fast-low-angle-shot (FLASH)-sequence). To measure the cutis one pixel was marked along the coil surface. The value of the whole lower leg = grey-values > 2 x background noise. Four tubes containing aqueous solutions with increasing Na^+^ concentrations (10, 20, 30, and 40 mmol/L NaCl) were positioned in a custom-made device positioned inside the coil just below the patient’s lower leg. Gray-scale measurements of the tubes served as calibration standards for ^23^Na-MRI of the skeletal muscle and skin by relating intensity to a concentration in a linear trend analysis. For H_2_O quantification, the 10 mmol/L NaCl tube served as a calibration standard for tissue water in a linear trend analysis defining a water content of approximately 1 liter water per liter volume. ROIs: 1 = cutis, 2 = triceps surae muscle, 3 = whole lower leg, 4–7 = calibration solutions, 8 = background noise.

An experienced radiologist interpreted the compartments of the lower leg, namely triceps surae muscle, cutis, and entire lower leg (see Fig 1). The different anatomical regions of interest were drawn guided by the anatomical image (T1-weighted fast-low-angle-shot (FLASH)-sequence). To measure the cutis one pixel was marked along the coil surface. The value of the whole lower leg = grey-values > 2x background noise. The interreader reliability was evaluated for ^23^Na measurements of 2 different probands that were assessed by four readers independently. Four tubes containing aqueous solutions with increasing Na^+^ concentrations (10, 20, 30, and 40 mmol/L NaCl) were positioned in a custom-made device positioned inside the coil just below the patient’s lower leg (see Fig 1). Gray-scale measurements of the tubes served as calibration standards for ^23^Na-MRI of the skeletal muscle and skin by relating intensity to a concentration in a linear trend analysis. Averaged images of four successively acquired gradient echo ^23^Na sequences were used to quantify Na^+^ concentration. For H_2_O quantification, the fat-suppressed inversion recovery sequence was evaluated. The 10 mmol/L NaCl tube served as a calibration standard for tissue water in a linear trend analysis defining a water content of approximately 1 liter water per liter volume. Since relative H_2_O changes were of interest, we used an arbitrary unit. Image analysis was performed using Image J software (Public Domain, Developer: Wayne Rasband).

We calibrated these techniques in earlier studies. Amputated lower limbs from subjects undergoing operations because of malignancy or diabetes were measured with ^23^Na-MRI. These limbs were desiccated (the difference between wet weight and dry weight was considered tissue water content) and ashed and measured with atomic absorption spectrometry, allowing us to show a very close correlation between ^23^Na-MRI signal and actual Na^+^ concentrations in muscle and skin [9, 10].

### Statistical analysis

Data are expressed as mean ± SD. Data were checked for normal distribution using the Shapiro-Wilk test. Data from ^23^Na/^1^H-MRI measurements and characteristics of the study population were analyzed by two-tailed student’s t-test for paired (before/after therapy) and unpaired (patients vs. controls) samples. *P*-values and confidence intervals (95% confidence level) were calculated using SPSS software (SPSS Statistics v20, IBM, Armonk, USA). Throughout the analysis, a two-sided *p*-value of less than 0.05 was considered statistically significant.

## Results

### Study population

Nine patients with acute heart failure were recruited (7 men/2 women; mean age 78 years; range, 58–87 years; mean blood pressure before therapy 125±23/73±14 mmHg, mean blood pressure after therapy 118±22/72±11 mmHg). The heart failure patients all had dyspnea and weight gain from their previous state. All patients had evidence of increased filling pressures based on physical examination, chest roentgenogram, and echocardiography. All had peripheral edema. The patients were receiving accepted medical treatment, including renin-angiotensin system blockade, beta-adrenergic blockade, and chronic diuretic therapy. All acute heart failure patients were scheduled to receive intravenous furosemide, based on their physician’s clinical judgment. The mean NT-proBNP blood level of the patients was 7892.11 ± 9688.10 (range: 682–27511) pg/mL before and 6314.60 ± 6953.22 (range: 824–22550) pg/mL after therapy (*p* = 0.3). We also recruited nine age- and sex-matched control subjects (7 men/2 women; mean age, 73 years; range 66–89 years; mean blood pressure 130±10/78±10 mmHg) who did not have heart failure and who were not ingesting any cardiovascular drugs. They were not treated with furosemide.

### ^23^Na/^1^H-MRI and plasma Na^+^

**Fig 2:**
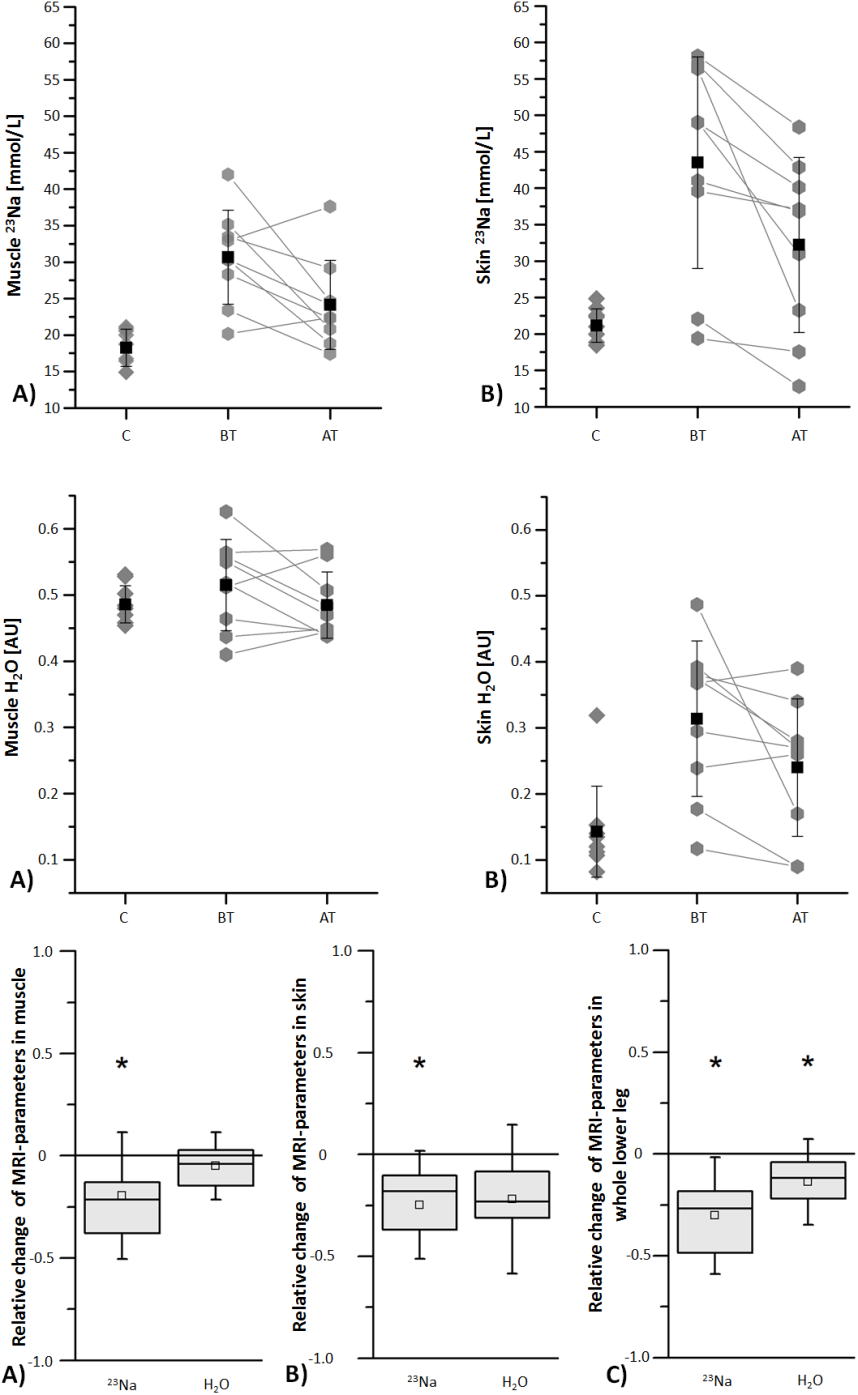
Na^+^ and H_2_O in muscle and skin in the lower leg of acute heart failure patients before and after diuretic treatment and of a healthy cohort. **Upper:** Mean Na^+^ concentrations determined by ^23^Na magnetic resonance imaging in muscle (A) and skin (B) significantly decreased during furosemide diuretic therapy (p ˂ 0.05 and p ˂ 0.01). Mean Na^+^ concentrations in muscle and skin of the lower leg of patients before and after therapy for acute heart failure were significantly higher compared to corresponding values of healthy subjects. **Middle:** Mean H_2_O contents determined by ^1^H magnetic resonance imaging in muscle (A) and skin (B) did not significantly decrease during furosemide diuretic therapy (*p* > 0.05). **Lower:** Box plots show the relative change ((AT-BT)/BT) of Na^+^ and H_2_O during furosemide diuretic therapy determined by magnetic resonance imaging in muscle (A), skin (B) and the whole lower leg (C) (upper horizontal line of box: 75^th^ percentile; lower horizontal line of box: 25^th^ percentile; horizontal bar within box: median; box within box: mean; upper and lower horizontal bar outside box: standard deviation). The asterisks indicate significant differences (*p* < 0.05). C = controls (healthy subjects), BT = before therapy, AT = after therapy, AU = arbitrary unit.

**Fig 3:**
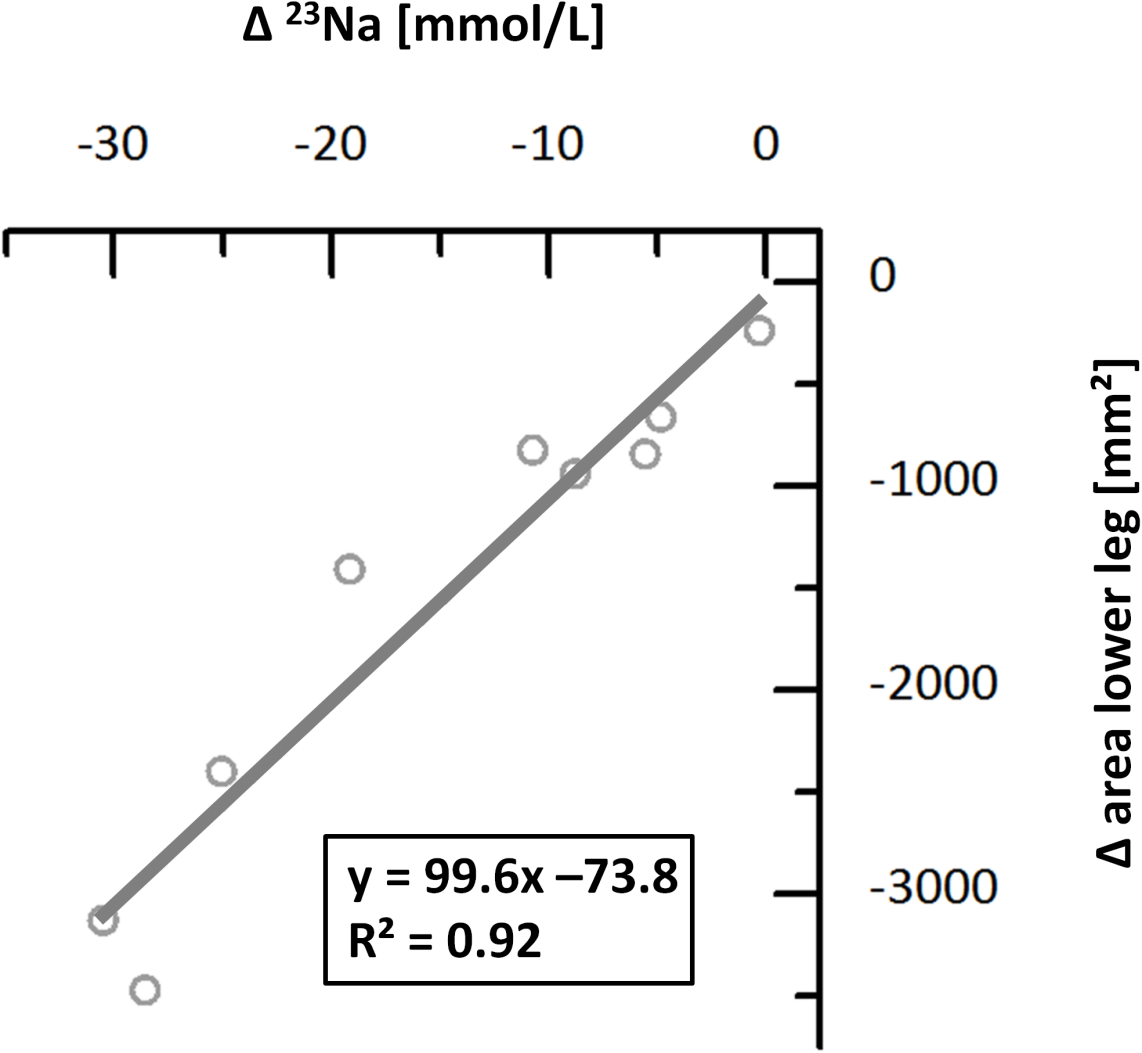
Correlation between the change of the Na^+^ concentration and the change of the area of the lower leg of acute heart failure patients during diuretic treatment.

^23^Na/^1^H-MRI methodology delivered satisfactory image quality in all examinations. Na^+^ concentrations were estimated based on our earlier findings [24–26]. Mean calculated Na^+^ concentrations by ^23^Na-MRI in the skeletal muscle and skin of the lower leg significantly decreased during furosemide diuretic therapy (see Fig 2 Upper). In two patients the Na^+^ concentration in the muscle increased during therapy. Na^+^ concentration before therapy was 30.7 ± 6.4 mmol/L in skeletal muscle and 43.5 ± 14.5 mmol/L in skin, respectively. After furosemide, Na^+^ concentrations were 24.2 ± 6.1 mmol/L in the skeletal muscle (*p* < 0.05) and 32.2 ± 12.0 mmol/L in skin (*p* < 0.01). Interestingly, water content measurements of muscle and skin did not differ significantly before and after diuretic therapy (*p* = 0.17 and *p* = 0.06) (see Fig 2 Middle). Box plots of the relative Na^+^ and H_2_O changes in muscle, skin, and the whole lower leg are shown in Fig 2 (Lower). The change of the Na^+^ concentration in the lower leg highly correlated with the change of the area of the lower leg (R^2^ = 0.92) during furosemide therapy deduced from ^1^H-MRI estimates (see Fig 3). Mean body weight loss during furosemide therapy was 3.6 ± 2.5 kg (*p* < 0.01).

The muscle Na^+^ concentration in patients before and after furosemide therapy was significantly higher than those of the healthy controls (18.3 ± 2.5 mmol/L; compared to patients before furosemide: *p* < 0.001; compared to patients after furosemide: *p* < 0.05). The same was true for skin (21.1 ± 2.3 mmol/L; compared to patients before furosemide: *p* < 0.01; compared to patients after furosemide: *p* < 0.05).

In contrast, plasma Na^+^ concentrations in the same patients did not change (138.5 ± 3.0 mmol/L before, 138.1 ± 4.3 mmol/L after, *p* = 0.6) and did not differ from those of the controls (138.8 ± 2.0 mmol/L; compared to patients before furosemide: *p* = 0.8; compared to patients after furosemide: *p* = 0.6). Furthermore, repetitive ^23^Na-MRI measurements of the same cross-section showed a high intra-method precision with an SD of 1.4% for both muscle and skin tissue (*n* = 9). Results are shown in Table 2.

**Table 2:** Demographic data and results.

	Patients		Control group	*P*-value (confidence interval)
*Gender*	7 men/2 women		7 men/2 women	
*Age [years]*	78 (range: 58–87)		73 (range: 66–89)	0.254 (-13.86–3.93)
	Before Therapy	After Therapy		
***Muscle Na*** ^***+***^ ***[mmol/L]***	30.7 ± 6.4	24.2 ± 6.1	18.3 ± 2.5	BT vs. AT: 0.026 (1.02–12.04); BT vs. C: 0.0001 (-17.32 –(-7.54)); AT vs. C: 0.017 (-10.60 –(-1.22))
***Skin Na*** ^***+***^ ***[mmol/L]***	43.5± 14.5	32.2 ± 12.0	21.1 ± 2.3	BT vs. AT: 0.008 (3.81–18.80); BT vs. C: 0.002 (-33.55 –(-11.96)); AT vs. C: 0.025 (-20.34 –(-1.75))
***Whole lower leg Na*** ^***+***^ ***[mmol/L]***	44.7 ± 15.3	29.9 ± 10.7	18.2 ± 2.5	BT vs. AT: 0.004 (6.20–23.45); BT vs. C: 0.001 (-38.27 –(-14.65)); AT vs. C: 0.011 (-19.93 –(-3.34))
***Muscle H***_***2***_ ***O [AU]***	0.52 ± 0.069	0.49 ± 0.050	0.486 ± 0.028	BT vs. AT: 0.168 (-0.02–0.08); BT vs. C: 0.266 (-0.08–0.03); AT vs. C: 0.955 (-0.04–0.04)
***Skin H***_***2***_ ***O [AU]***	0.31 ± 0.12	0.24 ± 0.10	0.14 ± 0.069	BT vs. AT: 0.064 (-0.01–0.15); BT vs. C: 0.002 (-0.27 –(-0.07)); AT vs. C: 0.033 (-0.18 –(-0.01))
***Whole lower leg H***_***2***_ ***O [AU]***	0.453 ± 0.101	0.386 ± 0.091	0.362 ± 0.042	BT vs. AT: 0.019 (0.02–0.12); BT vs. C: 0.030 (-0.17 –(-0.01)); AT vs. C: 0.487 (-0.10–0.05)
***Blood Na*** ^***+***^ ***[mmol/L]***	138.48 ± 2.96	138.09 ± 4.31	138.80 ± 2.00	BT vs: AT: 0.621 (-1.35–2.13); BT vs. C: 0.761 (-2.15–2.88); AT vs. C: 0.642 (-2.71–4.22)
***Blood NT-proBNP [pg/mL]***	7892.11 ± 9688.10	6314.60 ± 6953.22	NA	BT vs. AT: 0.295 (-1697.69–4852.80)
***Body weight [kg]***	85.9 ± 15.0	82.3 ± 14.9	82.4 ± 15.9	BT vs. AT: 0.003 (1.66–5.49); BT vs. C: 0.639 (-18.90–11.94); AT vs. C: 0.989 (-15.27–15.47)

Demographic data, [Na^+^] and H_2_O content in the muscle, the skin and the lower leg, Na^+^/NT-proBNP blood levels and body weight in patients with acute heart failure before and after diuretic (furosemide) therapy and on the control group are presented (mean ± SD). *P*-values (two-tailed student’s t-test) and confidence intervals (95% confidence level) are shown. BT = before therapy, AT = after therapy, C = controls (healthy subjects). AU = arbitrary unit. NA = not applicable.

**Fig 4:**
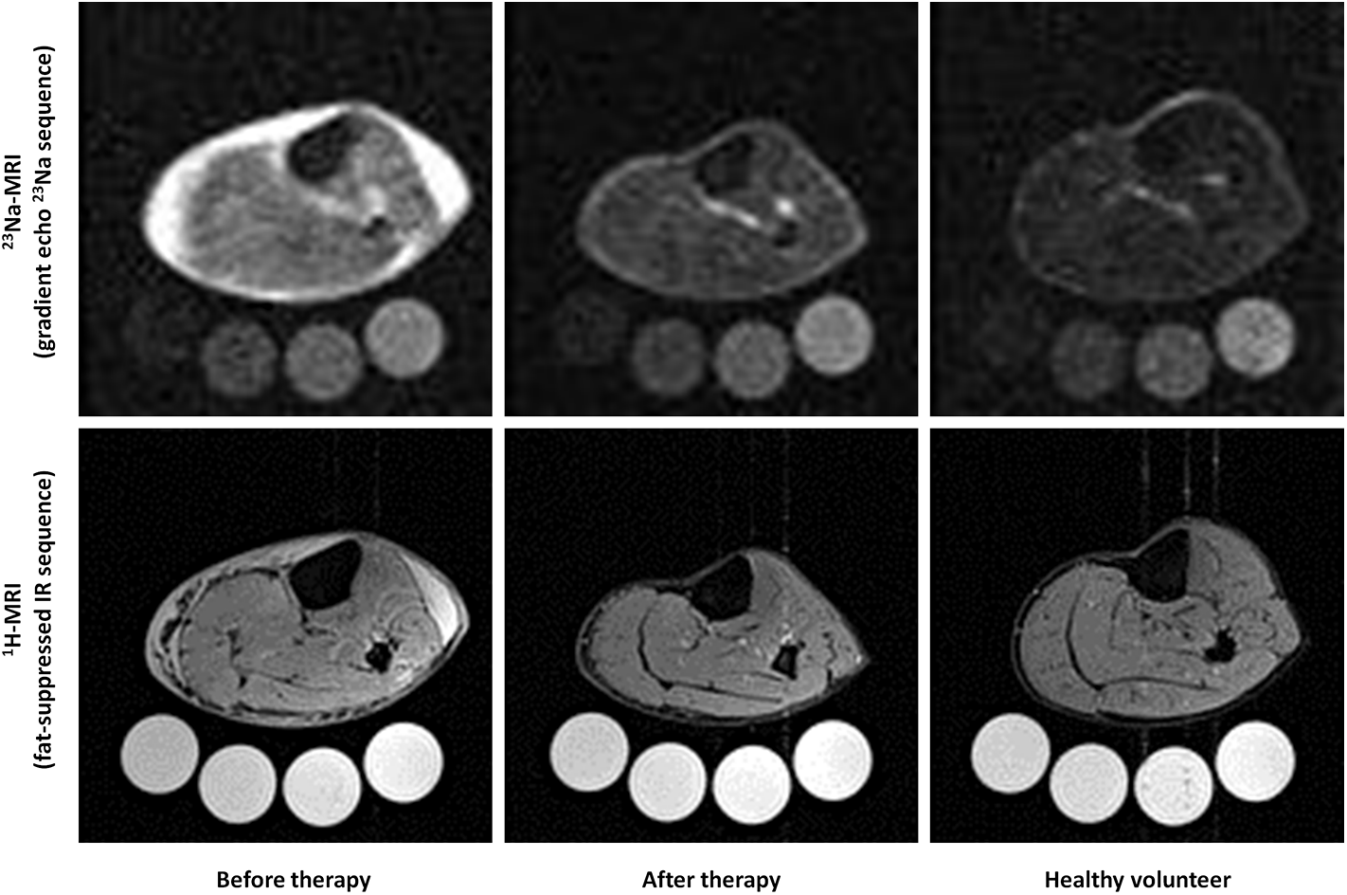
Exemplary ^23^Na/^1^H-MRI-images of a patient with acute heart failure before and after diuretic treatment and of a matched healthy subject. **Upper row:** ^23^Na scans (gradient echo ^23^Na sequence) of a 71-year-old patient before and after heart failure therapy. The amount of Na^+^ in skin and subcutaneous tissues, as well as in skeletal muscle was reduced, compared to the 10, 20, 30, and 40 mmol/L NaCl solutions (below). At far right is a normal 71-year-old control person with little sodium in skin and muscle. **Lower row:** The same patient before and after furosemide and the control subject viewed with conventional (^1^H) MRI (fat-suppressed inversion recovery (IR) sequence). The salt solutions appear white because of their water content.

^23^Na/^1^H-MR images from a 71-year-old patient with acute heart failure before and after furosemide diuretic therapy as well as representative ^23^Na/^1^H-MR images of a healthy 71-year-old volunteer are shown in Fig 4. A dramatic decrease in ^23^Na-MRI signal intensity can be noticed in the muscle and skin tissue, whereas the signal intensity of the 4 phantoms appears similar in both studies. Conventional ^1^H-MRI (fat-suppressed inversion recovery sequence) accompanying the ^23^Na-MRI studies allowed quantification of water content.

### Interreader reliability

The interreader reliability was evaluated for ^23^Na measurements of 2 different probands that were assessed by four readers independently. The standard deviations were 0.06/0.05 for the muscle, 0.15/0.27 for the skin and 0/0.11 for the whole lower leg (Table 3).

**Table 3:** Interreader reliability.

	Reader				SD
	1	2	3	4	
^**23**^ **Na-MRI proband 1**					
*Triceps surae muscle*	16.2	16.1	16.2	16.1	0.06
*Whole lower leg*	16.0	16.0	16.0	16.0	0
*Skin*	20.6	20.9	20.9	20.9	0.15
^**23**^ **Na-MRI proband 2**					
*Triceps surae muscle*	13.1	13.0	13.1	13.1	0.05
*Whole lower leg*	13.2	13.2	13.0	13.0	0.11
*Skin*	13.8	14.1	14.4	14.3	0.27

The interreader reliability was evaluated for ^23^Na measurements of 2 randomly selected probands which were assessed by four readers independently. Whole lower leg = grey-values > 2 x background noise. Skin = region where leg is positioned on the cylindrical phantom holders surface (one pixel-thickness = 3 mm). SD = standard deviation.

## Discussion

The important findings in our study are that ^23^Na-MRI shows accumulation of Na^+^ in muscle and skin in patients with acute heart failure. Diuretic treatment can mobilize this Na^+^ deposition. However, the water content of muscle and skin did not differ significantly before and after diuretic therapy.

Despite the fact that Na^+^ balance is assigned such an important role in the pathophysiology of acute heart failure, clinicians can only measure plasma concentrations, urinary Na^+^ excretion, or inquire about Na^+^ dietary intake. Visualizing Na^+^ in the body has not been possible. ^23^Na-MRI offers the opportunity to determine and measure Na^+^ deposition in the body. We show that Na^+^ can be measured in muscle and skin in patients with acute heart failure, that the levels in the patients are higher than in matched control subjects, and that the levels can be reduced with treatment. Although Na^+^ is also stored in bone, our data shown here and our earlier animal studies suggest that Na^+^ at that site is not very exchangeable and appears to play a negligible clinical role in patients with acute heart failure.

Another important finding from these observations is that Na^+^ can be stored non-osmotically, since the MRI images indicate Na^+^ accumulation without concomitant water accumulation. Like other researchers, we have shown that Na^+^ could be bound to GAG in skin [2, 27]. We have worked on the molecular mechanisms regulating Na^+^ deposition in skin. We documented local hypertonicity, with stimulation of TonEPB and a subsequent regulatory cascade that permits Na^+^ clearance from the skin [5–7]. When this clearance network is perturbed, salt sensitive hypertension results. These mechanisms were not yet investigated in animal models of heart failure, although such studies obviously should be done.

We are struck by the Na^+^ deposition in muscle. Our animal studies, in the desoxycorticosterone (DOCA) salt hypertension rat model, also indicated increased muscle Na^+^ concentrations. We have not yet investigated skeletal muscle in terms of Na^+^ storage, a lapse we intend to correct. Thus, we do not know about non-osmotic Na^+^ binding on the surface of skeletal muscle cells or conceivably inside these cells. In boys with Duchenne muscular dystrophy (DMD), ^1^H-MRI has revealed muscular edema before fatty degeneration. Weber et al. recently used ^23^Na-MRI to test the hypothesis that the edema is caused by an osmotic effect due to increased myoplasmic Na^+^ content rather than inflammation that would lead to extracellular edema [28]. They found that the DMD patients indeed had increased Na^+^ concentrations of 38.4 ± 6.8 mmol/L, compared to 25.4 ± 2.1 mmol/L in control subjects. The values that we report here for acute heart failure are even higher than those observed in the DMD patients. We have not determined TonEBP in skeletal muscle cells and have not measured intracellular or paracellular osmolality in this tissue. But since we did not observe muscle swelling, we must conclude that intracellular adjustments occur to deal with the situation. Na^+^/K^+^-ATPase is a ubiquitous enzyme present in higher eukaryotes responsible for the maintenance of ionic gradients across the plasma membrane. When intracellular Na^+^ levels rise, and after the activation of calcium-related signals, an intracellular Na^+^-sensing network is alerted that activates the Na^+^/K^+^-ATPase, which then expels the excess Na^+^ from the cytosol [29]. The sodium-induced kinase-1 (SIK1) is pivotal to that network and should be investigated in light of our findings.

Interestingly, diuretic therapy not only induced volume contraction in these patients accompanied by symptomatic relief, but also reduced muscle and skin Na^+^ concentration in stores. The value decreased in all patients in the skin and in 7 of 9 patients in the muscle, although the responses were variable. This variability suggests that patients differ in their propensity to release Na^+^ from muscle and skin. This patient-specific information could give clinicians an improved assessment of Na^+^ in heart failure patients and therefore might have a therapeutic implication. Interestingly, reductions in Na^+^ from stores were not accompanied by a similar reduction in water from the same sites. This state of affairs underscores the idea that Na^+^ was indeed stored non-osmotically, bound to proteoglycans.

In skin, we have shown that Na^+^ clearance is related to lymph-capillary density, which is in turn regulated by VEGF-C. A soluble VEGF-C receptor exists, termed soluble *fms*-like tyrosine kinase-4 (sFlt-4) [30]. Conceivably, the relationship of VEGF-C to sFlt-4 may influence the propensity of establishing lymph-capillary density in tissues and thereby influence Na^+^ removal from stores.

Our short-term study has limitations and suggestions for further work. Our study was necessarily observational and, aside from a single diuretic treatment, not longitudinal. We are also not able to speculate about any relationship between stores, dietary Na^+^ intake, or severity of disease. Furthermore, we used a rather long echo time (TE) of 2.07 ms in the ^23^Na-MRI sequence. Since the ^23^Na T2* signal decays bi-exponentially with a fast component (T2 fast) of 0.5 to 3 ms, it was postulated that pulse sequences to reliably measure signals from sodium should have an ultra-short echo time of less than 0.5 ms to minimize T2*-weighting [31]. Thus, with the used sequence parameters, the obtained signal may at least be partially influenced by other factors than sodium ions. However, we previously validated the utility of the applied ^23^Na-MRI methodology for quantitative analysis of tissue Na^+^ content in experimental animals and human subjects [25–27]. There is always a trade-off between the signal-to-noise ratio and spatial resolution in ^23^Na MR imaging. The accuracy of measuring Na^+^ concentration in the skin might be compromised by partial volume effects considering the in-plane resolution of 3 mm in this study for the gradient echo ^23^Na-MRI sequence. Therefore, we aimed to always place the lower legs in the same position (a customized cylindrical contact face was fitted inside the coil). Hence, the partial volume effects should be identical for different measurements. Furthermore, we demonstrated good precision for noninvasive detection of true differences in tissue Na^+^ content by ^23^Na-MRI methodology earlier [24]. We therefore conclude that trials on the changes of tissue Na^+^ during therapeutic procedures are possible by ^23^Na-MRI visualization. We are aware of male-female differences in Na^+^ deposition and could not pursue that issue here. We have no idea about Na^+^ stores in other ethnic groups. Nonetheless, we believe that our observations could give clinicians an improved assessment of Na^+^ in heart failure patients. Conceivably, these findings also have a therapeutic implication. For instance, other strategies such as mineralocorticoid receptor blockade, dietary modifications, or treatments directed at VEGF-C could be developed that might be more effective at reducing Na^+^ stores.

## Conclusions

^23^Na-MRI allows a reliable and non-invasive illustration and quantification of sodium concentration and distribution. ^23^Na-MRI shows accumulation of Na^+^ in muscle and skin in patients with acute heart failure. Diuretic treatment can mobilize this Na^+^ deposition. However, water content of muscle and skin did not differ significantly before and after diuretic therapy. Our study is too small and preliminary to show a relationship between Na^+^ mobilization and therapeutic responses; however, we are pursuing that hypothesis. ^23^Na-MRI enables new insights into sodium homeostasis that presumably lead to a better pathophysiologic comprehension. This may result in implications for diagnostic and therapeutic strategies.
